# Pay Pavilions of General Hospitals

**Published:** 1906-04-07

**Authors:** Ernest Collins

**Affiliations:** Chairman of the Council, Hampstead General Hospital.


### EDITOR'S LETTER-BOX.

Our Correspondents are reminded that prolixity is a great bar to publication, and that brevity of style and conciseness of statement greatly facilitate early insertion.]

### PAY PAVILIONS OF GENERAL HOSPITALS.

SIR,—The general attention which is at the moment directed to schemes for providing public institutions for patients able to pay for their support has brought to light a good deal of evidence of the efforts made to deal with this subject—as long ago as 1881 in the case of St. Thomas's Hospital, and 1884 in the case of Guy's Hospital.

Smaller institutions may also claim to have to a great extent solved the problem, and of these the Hampstead General Hospital was undoubtedly one of the first. Started as a Home Hospital in 1882, its special feature was that all patients were expected to contribute towards their support. It was found necessary later to provide free beds, the number of which has been increased now that the hospital has removed to the new building at Haverstock Hill. The experience of this institution goes to show that there is real need for beds for paying patients, and when in the near future the hospital is enlarged to 115 beds, it is intended to provide a special block with 20 beds for patients of this class, entirely separated from the free wards.

The problem is made more difficult by the want of uniformity in hospital methods. Applications for admission here, both to beds at the contributing rate of 12s. per week, and the private rooms at the rate of £3 3s. per week, have come from all parts of London. The former have necessarily been restricted to patients from the districts naturally served by the hospital, and as regards the latter, applications from the neighbourhood receive preference.

These charges serve as a working basis, variations being of course made in special circumstances, and the provision of the beds at the higher rate being made for the benefit of those only who cannot properly afford the charges in an ordinary Nursing Home.

I am given to understand that at least one of the London hospitals which have accommodation of this kind restricts the practice to consultants, but the course adopted in the Hampstead General Hospital, and which appears to be growing in favour, is that the patients shall be attended by their ordinary medical attendants, and that the necessary arrangements for the attendance of consulting physicians or surgeons shall be left in their hands.

I am, Sir, your obedient servant,
ERNEST COLLINS,
Chairman of the Council,
Hampstead General Hospital.

